# Technical optimization of spatially resolved single-cell transcriptomic datasets to study clinical liver disease

**DOI:** 10.1038/s41598-024-53993-2

**Published:** 2024-02-13

**Authors:** Brittany Rocque, Kate Guion, Pranay Singh, Sarah Bangerth, Lauren Pickard, Jashdeep Bhattacharjee, Sofia Eguizabal, Carly Weaver, Shefali Chopra, Shengmei Zhou, Rohit Kohli, Linda Sher, Omid Akbari, Burcin Ekser, Juliet A. Emamaullee

**Affiliations:** 1 Division of Abdominal Organ Transplantation and Hepatobiliary Surgery, Department of Surgery, Keck School of Medicine, University of Southern California, 1510 San Pablo Street, Suite 412, Los Angeles, CA 90033 USA; 2 Division of Gastroenterology, Hepatology and Nutrition, Children’s Hospital Los Angeles, Los Angeles, CA USA; 3 Division of Abdominal Organ Transplantation, Children’s Hospital Los Angeles, Los Angeles, CA USA; 4 Department of Pathology, Keck School of Medicine, University of Southern California, Los Angeles, CA USA; 5 Department of Pathology and Laboratory Medicine, Children’s Hospital Los Angeles, Keck School of Medicine, University of Southern California Los Angeles, Los Angeles, CA USA; 6 Department of Molecular Microbiology and Immunology, Keck School of Medicine, University of Southern California, Los Angeles, CA USA; 7 Division of Transplant Surgery, Department of Surgery, Indiana University School of Medicine, Indiana University, Indianapolis, IN USA

**Keywords:** Spatial transcriptomics, Single-cell spatial mapping, Biliary atresia, Cirrhosis, Liver disease, Translational research, Liver diseases, RNA sequencing

## Abstract

Single cell and spatially resolved ‘omic’ techniques have enabled deep characterization of clinical pathologies that remain poorly understood, providing unprecedented insights into molecular mechanisms of disease. However, transcriptomic platforms are costly, limiting sample size, which increases the possibility of pre-analytical variables such as tissue processing and storage procedures impacting RNA quality and downstream analyses. Furthermore, spatial transcriptomics have not yet reached single cell resolution, leading to the development of multiple deconvolution methods to predict individual cell types within each transcriptome ‘spot’ on tissue sections. In this study, we performed spatial transcriptomics and single nucleus RNA sequencing (snRNAseq) on matched specimens from patients with either histologically normal or advanced fibrosis to establish important aspects of tissue handling, data processing, and downstream analyses of biobanked liver samples. We observed that tissue preservation technique impacts transcriptomic data, especially in fibrotic liver. Single cell mapping of the spatial transcriptome using paired snRNAseq data generated a spatially resolved, single cell dataset with 24 unique liver cell phenotypes. We determined that cell–cell interactions predicted using ligand–receptor analysis of snRNAseq data poorly correlated with cellular relationships identified using spatial transcriptomics. Our study provides a framework for generating spatially resolved, single cell datasets to study gene expression and cell–cell interactions in biobanked clinical samples with advanced liver disease.

## Introduction

Biliary atresia (BA), the most common cause of end-stage liver disease in childhood, is clinically characterized as a perinatal progressive cholangiopathy affecting the bile ducts with a range of hypothesized etiologies^1^. Arising from a variety of potential sources, including targeted epithelial injury, defective embryogenesis, environmental toxins, and viruses, the immune landscape of BA is complex. Studies have shown CD4+ Th1 cell-mediated inflammation plays a critical role in the development of biliary obstruction^2^. Type-2 cytokines are known to induce hepatic fibrosis and cholangiocyte proliferation and have been a focal point of investigation in BA^3^. However, changes in gene expression profiles as well as the interplay between cholangiocytes and other cell types within the liver of patients with BA remain poorly defined.

Single-cell transcriptomic analyses created from cells in suspension including single-cell RNA-seq (scRNAseq) or single nucleus RNA-seq (snRNAseq) have provided mechanistic insights into liver disease in scarce clinical specimens at a resolution that was previously not possible^4–6^. These techniques have emerged as a valuable tool for the study of discrete cell phenotypes to enhance our understanding of disease states and identify potential therapeutic targets in liver disease. A normally functioning liver has preserved spatial architecture, including zones of discretely functioning hepatocytes, regions enriched with liver resident leukocytes, and areas designated for the production and excretion of bile, among others. Despite this intricate organization, to date, few studies have focused on the spatial aspects of liver disease pathogenesis, including processes involved in BA. MacParland et al. determined that non-inflammatory macrophage genes were localized in periportal regions while inflammatory macrophage genes were found closer to the central vein in healthy liver tissue^7^. Coupling spatial sequencing technologies, Melum et al. found that mesenchymal, monocyte, B-cell, and T-cell lineages within fibrotic regions were more prevalent than within parenchyma, suggesting that these subsets play important roles in hepatic fibrogenesis^8^. Incorporating spatial features into single-cell transcriptomics has the potential to examine important cellular interactions within the liver, allowing for the identification of more precise mechanisms of liver disease and highlights potential future therapeutic interventions.

A variety of spatial transcriptomic platforms are in development that enable analysis of gene expression patterns across histological regions of interest^9^. Spatial transcriptomics provide the additional benefit of describing the pathologic tissue landscape of diseases; however, best practices for the use of these techniques, particularly within the complex architecture of the liver, are unknown. Spatial transcriptomic data currently require integration with other sequencing modalities via computational deconvolution or spatial mapping methods to provide single-cell resolution and therefore gain novel insights into cellular features of disease processes with spatial manifestations^10,11^. Limitations include the cost associated with transcriptomic techniques and generation of highly dimensional and complex datasets, which can limit the volume of samples that can be processed and analyzed. Moreover, pre-analytical variables such as tissue storage and processing techniques can influence sequencing outputs, which may unintentionally confound results obtained from samples that are collected and stored in the clinical setting^12^.

The purpose of this study was to perform spatial transcriptomic analysis of the human liver, including samples of histologically normal liver and BA with advanced fibrosis. First, we employed two unique tissue preservation techniques within the same samples to determine the reproducibility of spatial transcriptomic data across experimental conditions. This is relevant to investigators who work with biorepositories containing frozen liver specimens. We integrated paired snRNAseq datasets to resolve the spatial profile and achieve single-cell resolution for 24 different liver cell phenotypes. We evaluated the correlation between predicted cell-to-cell interactions derived from ligand–receptor analysis of snRNAseq data to spatial transcriptomic outputs from the same tissue sample to establish the reliability of ligand–receptor (L–R) analysis to reflect true spatial motifs. We then focused our analysis on the spatially resolved molecular differences between normal and BA liver with advanced fibrosis. Together, these analyses provide a reference for performing spatial transcriptomic analysis on both healthy and fibrotic human liver tissue.

## Results

### Part 1: impact of different freezing techniques on spatial transcriptomic analysis of human liver samples

**Figure 1:**
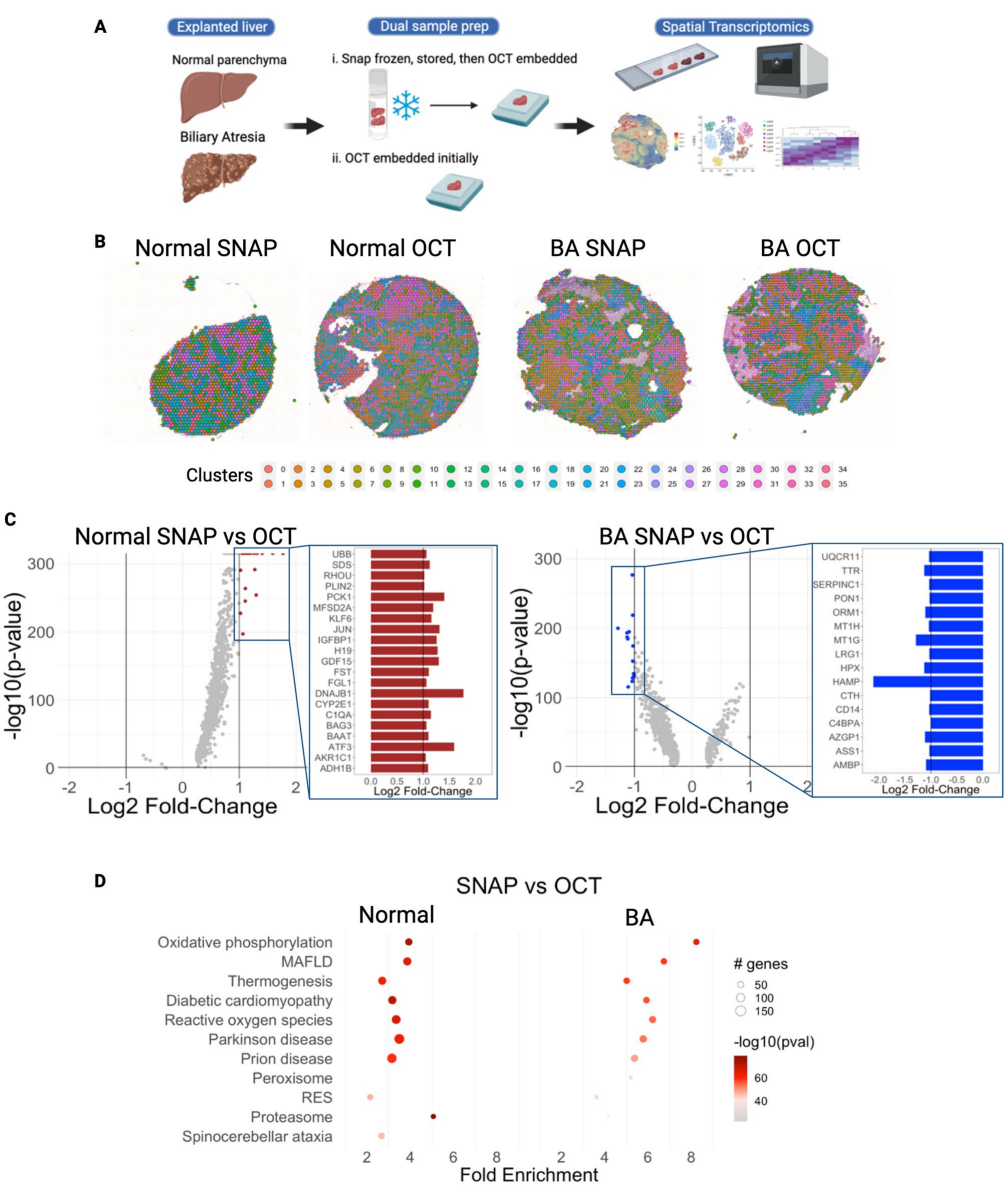
Liver tissue freezing technique impacts differential gene expression in oxidative stress pathways when using spatial transcriptomic analysis. (**A**) Samples of biliary atresia (BA) and normal parenchyma from hepatoblastoma liver were snap frozen, stored, embedded in OCT, and sectioned for ST (SNAP) or embedded in OCT and sectioned for ST (OCT). (**B**) Clustering of Visium spatial transcriptomics 55 μM spots. (**C**) Volcano plots of SNAP vs OCT gene expression in normal liver and BA with advanced fibrosis. Upregulated DE genes with a log2 fold-change greater than 1 are marked in red. Downregulated DE genes with a log2 fold-change less than -1 are marked in blue. Highlighted are the 21 upregulated genes in the normal liver and the 16 downregulated genes in the BA liver with advanced fibrosis. (**D**) Pathway analysis comparing SNAP and OCT preparation techniques in normal and BA with advanced fibrosis. *MAFLD* metabolic associated fatty liver disease, *RES* retrograde endocannabinoid signaling. (**A**) was created using BioRender, all other figures were created in R (version 4.2.2).

The constraints of clinical workflows, including the timing of biopsies and surgical procedures, can impact the ability to perform experiments using fresh liver samples. Also, many biorepositories bank samples of different disease states for future studies. In both instances, liver tissue is often snap frozen and stored at − 80 °C or in liquid nitrogen for future experiments^13^. The Visium platform enables spatial transcriptomic analysis of frozen tissue specimens, and the manufacturer recommends samples be frozen using OCT at the time of collection. Based on our prior work with scRNAseq of human liver, we hypothesized that liver tissue that was snap frozen and stored would generate different transcriptomic profiles than liver tissue that was initially frozen using OCT^14^. Fresh samples of normal liver tissue and BA with advanced fibrosis were collected and subject to two differing tissue preparation techniques at the time of collection: (i) snap freezing with storage in liquid nitrogen, followed by embedding in OCT at the time of retrieval from the freezer for transcriptomic analysis (‘SNAP’), versus (ii) embedding in OCT up front with storage in liquid nitrogen (‘OCT’) (Fig. 1A). A spatial map of gene expression for each 55 μm spot was generated for each sample using the Visium platform, and clustering algorithms were utilized to identify groups of spots with similar gene expression patterns (Fig. 1B). The analysis resulted in a diverse set of 36 clusters of spots representative of the spatial complexity in the liver in both SNAP and OCT conditions. Next, differential gene expression (DE) analysis was performed to compare SNAP and OCT preservation techniques in normal and BA with advanced fibrosis samples from the same patient. When comparing the two freezing techniques in normal liver, DE genes were largely upregulated, including activating transcription factor 3 (ATF3), DnaJ heat shock protein family member B1 (DNAJB1), and phosphoenolpyruvate carboxykinase 1 (PCK1) (fold change > 2.5), in SNAP relative to OCT (Fig. 1C, Supplementary Fig. S1A). When comparing BA with advanced fibrosis between freezing conditions (SNAP vs. OCT), the subset of genes that were DE were primarily downregulated, including hepcidin antimicrobial peptide (HAMP) and metallothionein 1G (MT1G) (fold change < − 2.2), in SNAP compared to OCT (Fig. 1C, Supplementary Fig. S1B). We next performed pathway analysis of DE genes, highlighting effects on the proteasome, oxidative phosphorylation, and reactive oxygen species in SNAP relative to OCT in both normal and BA samples, indicating that preservation technique likely impacts downstream transcript quality in these pathways (Fig. 1D). In BA with advanced fibrosis, genes associated with oxidative phosphorylation, metabolic associated fatty liver disease (MAFLD)^15^, and thermogenesis had even larger fold changes in SNAP relative to OCT than normal livers, indicating that diseased tissue with advanced fibrosis may be more sensitive to tissue processing techniques (Fig. 1D). Given these findings, only the OCT samples were used for further analysis.

### Part 2: integration of snRNAseq and spatial transcriptomic data via single-cell spatial mapping enables the creation of a spatially resolved single-cell atlas of the human liver

**Figure 2:**
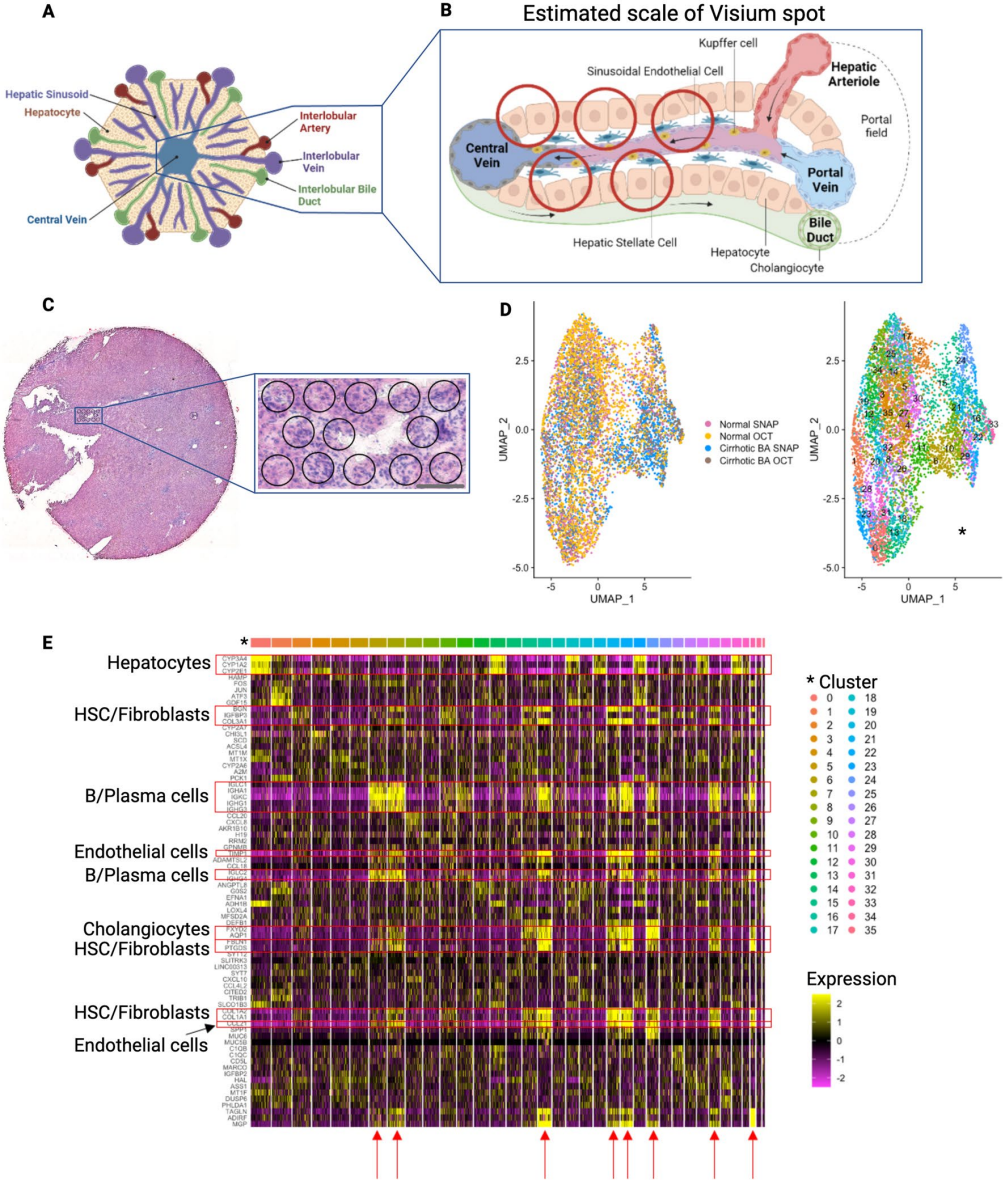
Clustering of spatial transcriptomic data spots yields mixed cellular phenotypes in human liver tissue. (**A**) Spatial organization of the liver portal tract showing individual cell types. (**B**) Liver sinusoid, with red circles corresponding to relative size of Visium spots (55 µm). Given the spatial organization of the liver with immune cells and cholangiocytes scattered throughout, a single spot is unlikely to resolve discrete cell populations. (**C**) H&E stained tissue section (normal liver) shows multiple nuclei (dark purple) per spatial spot. Scale bar = 100 μm. (**D**) UMAP visualization of spatial spots after integration of data obtained from all samples, labelled by sample on the left and by cluster on the right. (**E**) Differential gene expression within spot clusters. Red arrows highlight clusters with a mix in phenotype (Endothelial cells, HSC/fibroblasts, cholangiocytes and immune cells) affirming that clustering of these spatial spots cannot resolve individual cell populations. *HSC* hepatic stellate cells. (**A**) and (**B**) were created using BioRender, all other figures were created in R (version 4.2.2).

**Figure 3:**
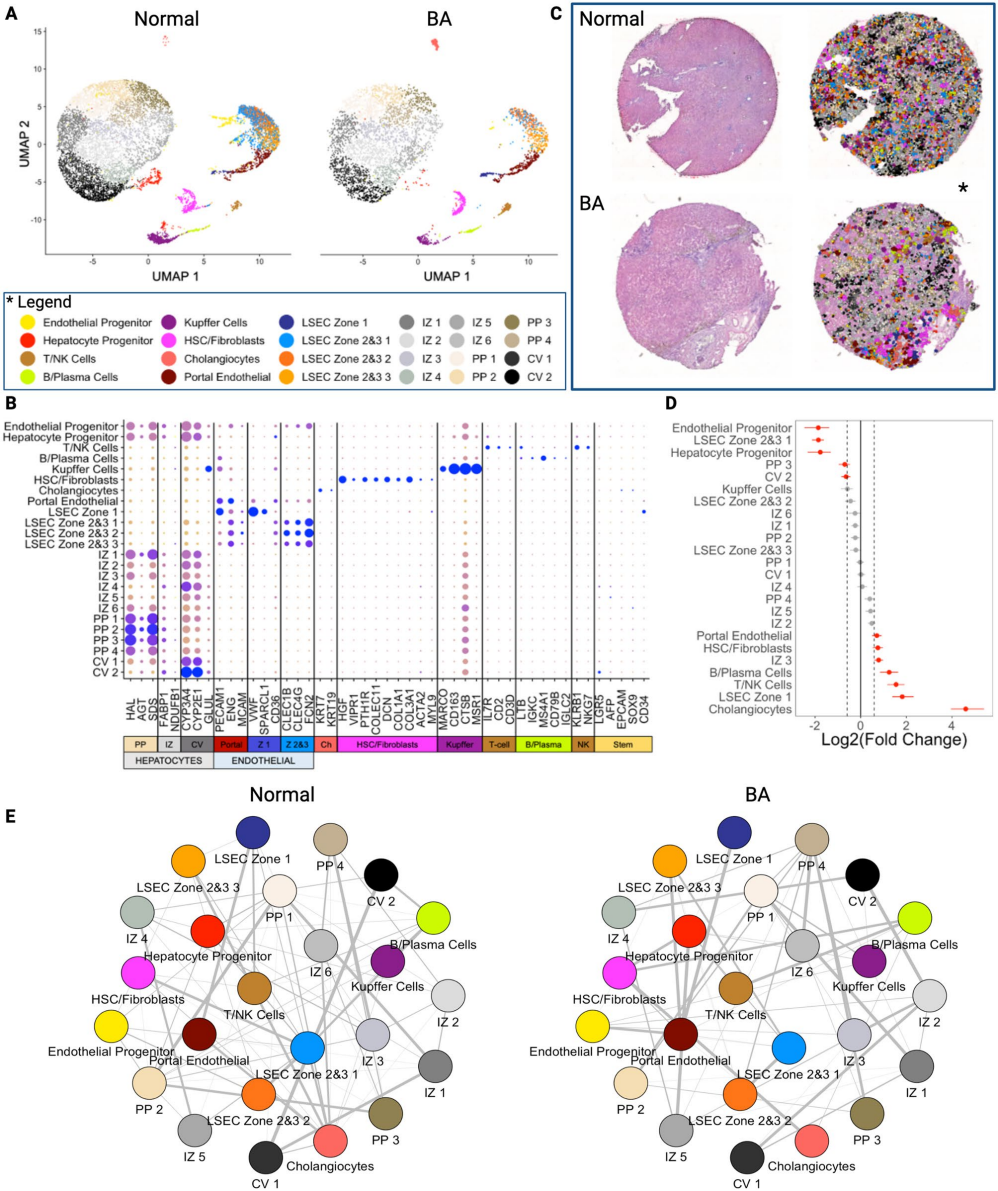
Single-cell spatial mapping of spatial transcriptomic data using matched snRNAseq data enables the creation of a spatially resolved, single-cell transcriptomic atlas with 24 discrete liver cell phenotypes. (**A**) Paired snRNAseq data were generated for each Visium sample (normal liver and BA liver with advanced fibrosis). UMAP visualization of single cells clusters by sample after cell labelling using established markers. (**B**) Gene expression heatmap of individual cell types with corresponding cell-type markers. (**C**) Spatial visualization of single-cell spatial mapped dataset. The left column represents the tissue slide after H&E principal staining and the right column includes overlay of single cell types after single-cell spatial mapping. The regions with absent cells represent regions with no spatial spots as they were discarded due to poor quality during our quality control steps. (**D**) Cell proportion comparison between normal and BA with advanced fibrosis using permutation testing (red signifies FDR < 0.05 and abs(Log2(Fold Change)) > 0.58). BA liver with advanced fibrosis has higher proportions of cholangiocytes, LSEC zone 1, T/NK cells, B/plasma cells, IZ 3, HSC/Fibroblasts, and portal endothelial cells. Normal liver has higher proportions of endothelial progenitor cells, LSEC zones 2&3 1, hepatocyte progenitor cells, PP 3 cells, and CV 2 cells. (**E**) Correlation plots of cell clusters for both normal and BA with advanced fibrosis samples using single-cell spatially mapped data. Line thickness correlates to spatial associations between two cell types. Overall, strength of spatial association is decreased in BA with advanced fibrosis. When looking closer at the individual interactions, BA with advanced fibrosis sees an increase in the strength between endothelial cells, immune cells, and between HSC/Fibroblasts with B/Plasma cells and cholangiocytes. *CV* central-venous hepatocytes, *HSC* hepatic stellate cells, *IZ* interzonal hepatocytes, *LSEC* liver sinusoidal endothelial cells, *NK* natural killer cells, *PP* periportal hepatocytes. All figures were created in R (version 4.2.2).

Liver tissue is highly spatially organized with portal triads and central veins connected by sinusoids (Fig. 2A). The Visium spatial transcriptomic platform generates output ‘spots’ of 55 μm diameter, which can represent 1–10 cell types per spot (Fig. 2B). Superimposing the spatial spots on the actual H&E stained tissue section confirms that a single Visium spot can represent multiple nuclei (Fig. 2C). When attempting to identify cell types based solely on Visium spatial transcriptomics data (Fig. 2D), we see multiple cell types present in each cluster, as shown in the heatmap in Fig. 2E, affirming that clustering of spatial spots cannot resolve individual cell populations. Recent advances in informatics methods have been developed to map single cell transcriptomes from scRNAseq or snRNAseq outputs onto spatial transcriptomic datasets. There are two general approaches: identification of the percent of each cluster or cell type in a spatial spot (CARD^16^ or Seurat^10^), also known as deconvolution, versus mapping a single cell type to a specific spot (Celltrek^17^). We implemented both techniques, CARD and Celltrek (Supplementary Fig. S2). Given the complexity of liver tissue and the desire to understand spatial relationships between individual cell types and their transcriptomes, rather than just their location, we employed the second approach to map single cell types to Visium spots using Celltrek for downstream analysis since it improved output interpretability. To perform single-cell spatial mapping, we first generated matched snRNAseq datasets from both normal and BA with advanced fibrosis samples. Unsupervised clustering was used to generate 24 clusters within the snRNASeq data, and established cell lineage markers were used to annotate individual cell types (Fig. 3A,B)^18^. Relative shifts in cell proportions were evaluated between normal and BA with advanced fibrosis samples and revealed that BA tissue had increased cholangiocytes, liver sinusoidal endothelial cells (LSEC) zone 1, T/NK cells, B/plasma cells, hepatic stellate cells (HSC)/fibroblasts, and portal endothelial cells (Fig. 3D). BA with advanced fibrosis had also decreased endothelial progenitor cells, LSEC zones 2&3, and hepatocyte progenitor cells. These annotated snRNAseq datasets were then used to spatially map single cells onto the spatial OCT samples (Fig. 3C). Using these spatially resolved single-cell data, colocalization analysis was completed to identify spatial distribution of different cell types in the tissue (Fig. 3E). This revealed a loss of overall complexity in the cellular interactions in BA with advanced fibrosis. Looking closer at individual colocalization interactions, BA with advanced fibrosis is associated with an increase in the colocalization strength between different types of endothelial cells and hepatocytes as well as between HSC/fibroblasts, cholangiocytes, and immune cells. In normal liver tissue, immune populations and HSC interact more with LSEC and hepatocyte populations.

**Figure 4:**
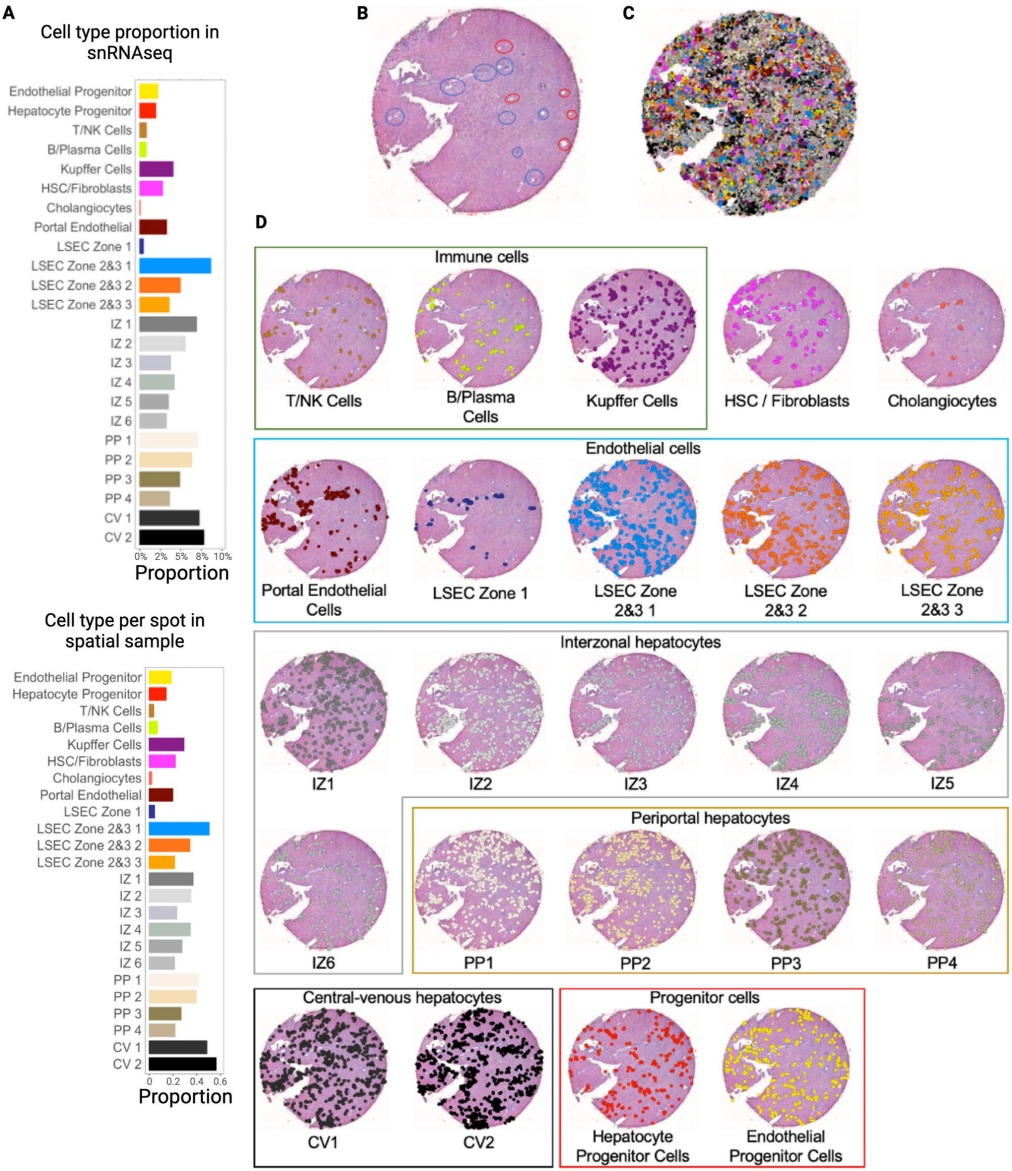
Single-cell spatial mapping of spatial transcriptomic data enables identification of individual cell types in normal liver. (**A**) Proportion of each cell type in the snRNAseq sample and proportion of cells per spot of each cell type in the spatial sample. The distribution in both samples is similar, affirming that the proportion is taken into consideration during the single-cell spatial mapping process. (**B**) H&E stained tissue section after annotation by a pathologist, with red circles corresponding to central veins and blue circles to periportal regions. (**C**) Spatial visualization of single-cell clustering after single-cell spatial mapping overlayed on H&E stain. (**D**) Overlay of single-cell clustering after single-cell spatial mapping visualized on H&E section for each cell type. *CV* central-venous hepatocytes, *HSC* hepatic stellate cells, *IZ* interzonal hepatocytes, *LSEC* liver sinusoidal endothelial cells, *NK* natural killer cells, *PP* periportal hepatocytes. All figures were created in R (version 4.2.2).

**Figure 5:**
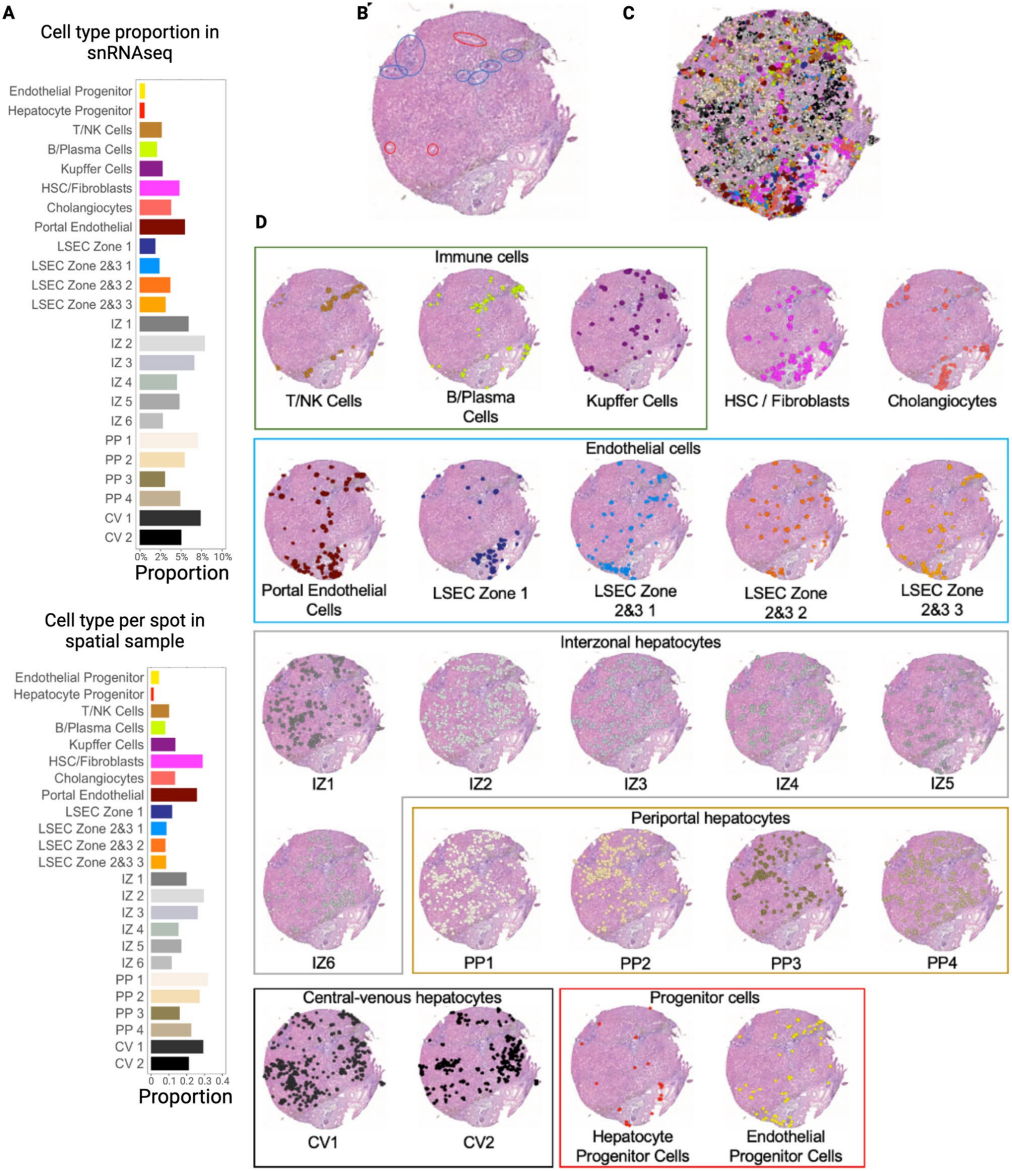
Single-cell mapping of spatial transcriptomic data enables identification of individual cell types in BA with advanced fibrosis tissue. (**A**) Proportion of each cell type in the snRNAseq sample and proportion of cells per spot of each cell type in the spatial sample. The distribution in both samples is similar, affirming that the proportion is taken into consideration during the single-cell spatial mapping process. (**B**) H&E stained tissue section after annotation by a pathologist, with red circles corresponding to central veins and blue circles to periportal regions. (**C**) Spatial visualization of single-cell clustering after single-cell spatial mapping overlayed on H&E stain. The regions with absent cells represent regions with no spatial spots as they were discarded due to poor quality during our quality control steps. (**D**) Overlay of single-cell clustering after single-cell spatial mapping visualized on H&E section for each cell type. *CV* central-venous hepatocytes, *HSC* hepatic stellate cells, *IZ* interzonal hepatocytes, *LSEC* liver sinusoidal endothelial cells, *NK* natural killer cells, *PP* periportal hepatocytes. All figures were created in R (version 4.2.2).

Each cell type was projected onto the spatial transcriptomic output and visualized relative to H&E staining to demonstrate density and spatial distribution in normal liver (Fig. 4) and BA with advanced fibrosis tissues (Fig. 5). To increase our confidence in the single-cell spatial mapping approach, we observed that the proportion of cells identified in each cell type cluster in the single-cell spatially mapped data mirrored the snRNAseq data in both normal and BA liver tissues. In normal liver, the highest proportion of cells belonged to the LSEC zones 2&3 subcluster 1 cluster and the central-venous hepatocyte clusters (Fig. 4A). This is also seen in the visualization of the single-cell spatially mapped data on the tissue sample (Fig. 4C,D). Additionally, the annotated cells can be compared to pathologist-labeled periportal and central venous regions (Fig. 4B). While not spatially exclusive, cells identified in the portal endothelial cell gene expression cluster and the LSEC zone 1 cluster fall in the periportal regions (Fig. 4D). In BA with advanced fibrosis, cells identified in the interzonal 2 cluster were the largest proportion of cells (Fig. 5A), mirroring the distribution of cells in the single-cell spatially mapped data (Fig. 5C,D). When compared to microanatomy annotations (Fig. 5B), the immune cell types in BA were usually in periportal areas (Fig. 5D) whereas their spatial distribution was more diffuse in normal liver (Fig. 4D).

### Part 3: spatial transcriptomic data augments ligand–receptor analysis in human livers

**Figure 6:**
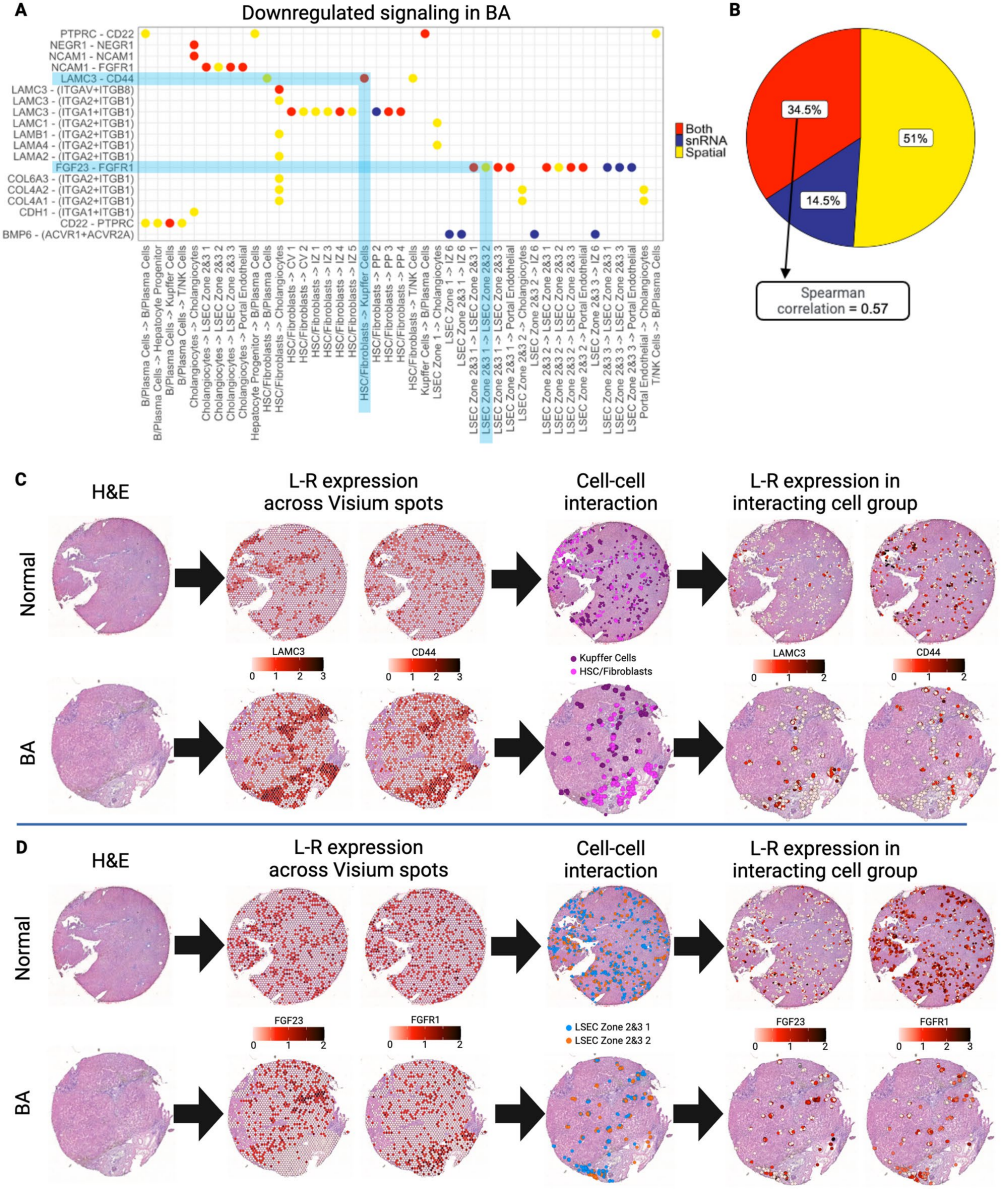
Cluster interaction analysis illustrates the difference in ligand–receptor analysis in BA with advanced fibrosis when comparing snRNAseq and spatially resolved transcriptomic data. (**A**) L–R pairs in interacting cell types show a decrease in signaling in BA liver with advanced fibrosis when compared to normal liver tissue. The three distinct colors represent whether interactions were only downregulated in snRNAseq data (blue), only in the spatially resolved transcriptomic data (yellow), or in both datasets (red). Two representative cell–cell interactions are shaded in light blue. The first indicates downregulated L–R signaling present in both datasets (red dot, spatially visualized in panel **C**), and the second one indicates downregulated L–R signaling present only in the single-cell spatially mapped dataset (yellow dot, spatially visualized in panel **D**). (**B**) Only 34.5% of downregulated L–R signaling pairs are present in both snRNAseq and spatial transcriptomic datasets (Spearman correlation = 0.57). 51% of downregulated L–R signaling pairs are only present in the spatial data, while 14.5% are only present in the snRNAseq data. *CV* central-venous hepatocytes, *HSC* hepatic stellate cells, *IZ* interzonal hepatocytes, *LSEC* liver sinusoidal endothelial cells, *NK* natural killer cells, *PP* periportal hepatocytes. All figures were created in R (version 4.2.2).

Next, L–R analysis was performed, first on the snRNAseq data and then on the single-cell spatially mapped transcriptomic data. Overall, the L–R pairs among interacting cell types showed a decrease in signaling in BA with advanced fibrosis when compared to normal liver, in either the snRNAseq data, spatial transcriptomic data, or both datasets (Fig. 6A). No significantly upregulated L–R interactions were found in BA with advanced fibrosis when compared to normal liver when analyzing the snRNAseq samples. Overall, 51% of the L–R interactions were downregulated in only the spatial transcriptomic dataset, whereas 14.5% were downregulated only in the snRNAseq dataset with the remaining 34.5% downregulated in both (Fig. 6B). This highlights that the snRNAseq data alone may not be sufficient to understand potential L–R interactions in a liver tissue sample.

Comparison with single-cell spatially mapped data adds additional information regarding communication pathways. For example, the Laminin Subunit Gamma-3 (LAMC3)-CD44 interaction present between HSC/fibroblasts and Kupffer cells is downregulated in BA with advanced fibrosis in both the snRNAseq and spatial transcriptomic data (Fig. 6C). In this instance, single-cell spatial mapping analysis adds confidence to the results from the snRNAseq L–R analysis that BA with advanced fibrosis samples show potential interference with the communication pathway between these two cell types; specifically between LAMC3, a major component of basement membranes, and CD44, a cell surface receptor that has been implicated in fibrotic and wound healing processes^19–21^. In contrast, the Fibroblast Growth Factor-23 (FGF23)—Fibroblast Growth Factor Receptor-1 (FGFR1) interaction from LSEC zones 2&3 subcluster 1 to subcluster 2 is only downregulated in BA spatial transcriptomic data (Fig. 6D). For this interaction, the spatial transcriptomic data added information not previously seen when analyzing snRNAseq data. This suggests that within LSEC zones 2&3 of BA tissue, there is a distinctively spatial dysregulation of the FGF23-FGFR1 pathway, which has been implicated in both liver metabolism and the development of fibrosis^22^. As FGF23 is known to be an antagonist of myofibroblast activity, a decrease in FGF23 signaling, as observed in BA tissue, leads to increased myofibroblast activity and therefore fibrosis progression^23^.

When comparing L–R interactions downregulated in both datasets, we see a low correlation in communication strength (Spearman correlation of 0.57). Looking at the relative change in communication strength in these interacting cell groups further corroborates that there is a difference in the L–R findings of snRNAseq and single-cell spatially mapped data (Supplementary Fig. S3A). This highlights that the snRNAseq data alone are not sufficient to understand potential L–R interactions in a liver tissue sample, especially given that these findings cannot be validated in snRNAseq data. Predicted L–R interactions from snRNAseq are enhanced by comparing single-cell spatially mapped transcriptomic data; this not only adds information on communication pathways, but the findings can be validated by projecting L–R pairs onto the actual tissue.

While SNAP spatial samples were not used in downstream analysis, we did compared L–R interaction results between the two different techniques for each patient (Supplementary Fig. S3B). Overall, 25% of the L–R interactions were downregulated in only the OCT dataset and 35% in only the SNAP dataset, while 40% were downregulated in both (Supplementary Fig. S3C). The difference in downregulated interactions between the two samples is not surprising given that we already saw a difference in transcriptional changes when individually comparing the two freezing techniques in normal and BA with advanced fibrosis and that the tissue blocks are different, resulting in differences in the number of portal triads, central veins, etc. (Fig. 1). Despite using different tissue blocks and the impact of freezing techniques on some gene expression pathways, the communication strength of the overlapping L–R signaling pairs between samples from the same patient was high when compared to the snRNAseq vs spatial comparison (Spearman correlation coefficient of 0.98 versus 0.57). This increase in the correlation of spatial interactions further supports comparing and confirming L–R analysis findings with spatial transcriptomics where possible.

## Discussion

This study aimed to investigate the workflow of spatial transcriptomic analysis in clinical liver specimens, encompassing tissue preservation, data preprocessing, and downstream analysis. We first examined two different tissue processing techniques, snap-frozen in liquid nitrogen or cryopreserved in OCT blocks, and observed that pre-analytic variables can impact spatial transcriptomic outputs. In both normal liver and BA with advanced fibrosis tissues, DE analysis results suggest there are alterations in cellular responses due to differences in tissue processing. With the samples from the different freezing techniques being patient-matched in both comparisons, it is likely that the additional freezing step in the snap-frozen tissue is the reason we see these transcriptional changes. In fact, snap-frozen tissue was more likely to have alterations in cell stress pathways including oxidative phosphorylation, thermogenesis, and reactive oxygen species, which is consistent with our prior study integrating multiple liver scRNAseq datasets demonstrating that pathways involved in protein synthesis, cell death, and apoptosis signaling are affected by tissue processing^14^.

When comparing the pathway analysis results, we observe that the fold enrichment in BA liver is larger compared to normal liver for the same pathways. Given that BA liver is already diseased tissue with many DE genes compared to normal liver, while also being more sensitive to tissue processing and other preanalytical variables due to the advanced fibrosis, the baseline of comparison between these two tissues when comparing the two freezing techniques is different. At baseline, BA liver likely has stress pathways that are already upregulated compared to normal liver. Adding additional stress in the freezing process—as is the case with snap-frozen tissue—increases the already active stress pathway even further. It is therefore not surprising that BA liver has higher enrichment scores for cell stress pathways.

Our prior work and the current study highlight that the choice of experimental method does impact most gene expression pathways in downstream single-cell transcriptomic analysis of clinical liver specimens. With regards to spatial transcriptomic outputs, OCT was superior to SNAP, as it did not upregulate cellular stress pathways to the same extent, thus preserving the underlying transcriptomics more accurately in both normal and fibrotic liver tissues.

Next, we explored how single-cell spatial mapping using paired snRNAseq data enables the creation of a single-cell, spatially resolved transcriptomic dataset using banked clinical liver samples. Single-cell spatial mapping is a crucial step in analyzing spatial transcriptomic datasets, as each spatial spot contains multiple cells and snRNAseq data lacks spatial information. We utilized snRNAseq data to define 24 different liver cell phenotypes and explored two popular approaches: CARD^16^ and CellTrek^17^ (Supplementary Fig. S2). While CARD, a deconvolution technique, provided information on the percentage of each single nuclei cell type within each spot, visually interpreting this data with 24 cell phenotypes was challenging, making it difficult to confirm neighborhood relationships between cell types. Indeed, a recent publication examining BA liver used the Spotlight technique to generate a pie chart to visualize the proportions of cell types on each Visium spot, limiting the analysis of potential interactions between cells of interest^24^. In contrast, mapping snRNAseq data onto the Visium spots using CellTrek, a single-cell spatial mapping technique, provided a more visually intuitive figure, enabling better validation of biological accuracy for specific cell types. Visual confirmation of single-cell spatially mapped cell types to pathologist-labeled liver tissue samples substantiated this approach. For instance, LSEC zone 1 cells were primarily in the pathologist-identified periportal regions, as expected (Figs. 4 and 5). In addition, single-cell spatial mapping enabled additional downstream analysis. CellTrek specifically provided a spatial colocalization analysis that highlighted the spatial relationship between each cell type cluster. This type of spatial analysis can be used for both validation of biological accuracy via the confirmation of known spatial relationships, as well as the discovery of new spatial relationships. Our analysis highlights how spatial transcriptomics can be used to validate potential cellular interactions derived from L–R analysis, a technique frequently used to explore scRNAseq and snRNAseq data. Although our sample size is small, predicted L–R interactions from matched snRNAseq outputs poorly correlated with interactions using our single-cell spatially mapped transcriptomic data in both healthy and fibrotic tissue (Spearman correlation = 0.57, Fig. 6), while outputs between the two different spatially resolved transcriptomic datasets—OCT and SNAP—from unique tissue blocks from the same liver sample were highly correlated (Spearman correlation = 0.97, Supplementary Fig. S3B,C). This suggests that investigators should be cautious when interpreting L–R interaction results from an output that cannot be validated, such as is the case for snRNAseq data, as it can infer spatial relationships that may not reflect the actual biology of pathologic processes in clinical samples. Additionally, as new techniques to assess L–R interactions are emerging, such as stLearn^25^, these should be evaluated as potential alternatives when analyzing spatial samples, which we plan on doing in future studies.

While not the central focus of this study, our analysis revealed spatially relevant transcriptomic changes in BA with advanced fibrosis. First, BA tissue had a quantitative increase in immune cells, both T/NK cells and B/plasma cells, as well as increased HSC/fibroblast cells. This is consistent with a prior study that used scRNAseq in BA, identifying a mixed immune and fibrotic response to damaged livers^24^. This is supported by the spatial colocalization analysis that demonstrated increased strength between immune populations in BA—which suggests that immune cells are responding to injury within the portal triad—as well as an increased colocalization strength between immune cells and HSC/fibroblasts. In normal liver tissue, we expect fewer immune populations present in the tissue and, those that are present, will be rather scattered and not localized to a specific area. This is supported by the cell–cell colocalization analysis where we see immune cells in proximity to a variety of other cell populations, including LSEC and hepatocytes. Additionally, BA tissue showed a decrease in progenitor cells and LSEC zones 2&3. The decrease in endothelial and hepatocyte progenitor cells is expected with liver damage seen in end-stage BA. LSEC zones 2&3 cells are important spatially as zones 2&3 are histologically furthest from the portal triad and most susceptible to injury. The decreased proportion of LSEC zones 2&3 suggests either that these cells are dying and replaced by fibrosis and/or that, in the injury process, these cells can no longer be identified as LSEC zones 2&3 based on their gene expression profile. As Su et al. also demonstrated that LSEC zones 2&3 tend to retain their identities in snRNAseq cirrhosis data, it is likely that the decrease in cells is secondary to fibrotic development^26^. In addition, these cells had a downregulation of L–R signaling from the LSEC zones 2&3 subcluster 1 to subcluster 2 (Fig. 6). This suggests that there is a spatial dysregulation of cellular signaling within these zones, potentially contributing to unregulated fibrosis progression. Furthermore, there was a downregulation of the LAMC3–CD44 interaction from the HSC/fibroblasts to the Kupffer cells in the BA tissue. This is interesting as other studies have highlighted CD44 in the inflammatory and fibrotic development of liver cirrhosis^27,28^. While this finding may be due to the limitations of this study as discussed below, it is interesting to consider if previously known biomarker pathways of liver disease can be influenced by the addition of spatial analysis.

Various limitations should be considered in this study. First, its small sample size decreases the generalizability of the findings. Additionally, previous research has shown that the transcriptome is not equivalent to the proteome of the organism^29^. In our transcriptomic based study, we cannot assume that the RNA analysis directly translates to the resulting phenotype of each liver cell. Moreover, the comparison of the two experimental techniques was performed using spatial transcriptomics. Despite observing a high correlation in the L–R analysis results between the two different freezing techniques (Spearman correlation = 0.97), the transcriptomes may be different between the samples of the same patient given that it is not possible to govern which cells are localized from the 55 μM spots. Finally, snRNAseq data are mapped onto spatial transcriptomic data based on the similarity between the gene profile of a given spatial spot and the gene profile of a single nucleus. Raw Visium data have gene expression profiles in each spot, which can be replaced by single cell outputs following spatial mapping. Thus, after single-cell spatial mapping, the gene expression profile of each spatial spot will be replaced with the gene expression of the single-cell data mapped to it; if more than one cell is mapped to one spot, this spatial spot will have the gene expression of each individual cell. Raw marker expression should therefore be analyzed for each Visium spot before single-cell spatial mapping to retain the integrity of the Visium output. After single-cell spatial mapping, marker expression becomes dependent on single-cell data. The accuracy of cell type labels and cell–cell communication used in the L–R analysis of the spatial data is therefore directly linked to the accuracy of single-cell mapping and cell labeling of the snRNAseq data.

Spatial transcriptomics will continue to expand our understanding of clinical liver disease. In this study, we have highlighted important technical caveats and provided a roadmap for working with these complex datasets to explore the pathogenesis of end-stage BA at single-cell resolution. Using this approach, we observed that BA was characterized by a loss in cell–cell interaction complexity, dysregulation of FGF23–FGFR1 signaling, and loss of LAMC3–CD44 interactions between hepatic stellate cells and Kupffer cells. Single-cell spatial mapping of transcriptomic data is a relatively new technique, and our study confirms that high-quality, single-cell resolution spatial transcriptomic techniques can be reliably employed to study both normal and diseased biobanked human liver samples.

## Methods

This study was begun after obtaining approval from the Institutional Review Board at the University of Southern California (CHLA-19-00177). All research was performed in accordance with the Declaration of Helsinki, and informed consent from legal guardians (patients were under 18 years of age at enrollment) was obtained for all tissue and clinical data obtained for this study.

### Clinical specimens

Human fresh liver tissue specimens were collected from explants from two patients undergoing liver transplant. For the purposes of this study, an area of histologically normal liver obtained from a patient undergoing transplant for hepatoblastoma was designated as ‘normal’ liver tissue while liver tissue with advanced fibrosis was obtained from a patient undergoing transplant for end-stage BA (confirmed via Hematoxylin and Eosin (H&E) staining and review by expert pathologists). Each patient had two samples, one for each experimental condition. Fresh liver was placed in sterile saline and transported to the lab on ice. Samples were diced to approximately 0.5 cm cubes, and portions were either snap-frozen in liquid nitrogen (SNAP) or cryopreserved in OCT blocks (OCT). These spatial transcriptomics samples were stored in liquid nitrogen for less than a month before processing. On the day of sample processing for transcriptomic analysis, SNAP specimens were blocked using OCT without prior thawing. Both sets of OCT-embedded specimens were then transported on dry ice to the Molecular Genomics Core of the Norris Comprehensive Cancer Center of USC for Visium Spatial Gene Expression Assay (10× Genomics).

### Transcriptomics

Spatial transcriptomic profiling was performed using the 10× Genomics Visium CytAssist. From the same sample block, scrolls were sectioned, and a sequential slide stained with H&E was reviewed by a liver pathologist to select regions of interest including portal, periportal, and centrivenular areas. RNA extraction from the tissue scrolls was performed using the Qiagen RNeasy kit, and RNA quality control was assessed using the Agilent BioAnalyzer. The sample block was trimmed and sectioned at 10 μm onto charged slides and transferred to the spatial profiling slides through the CytAssist instrument and proceeded into library preparation. Quality control of the libraries was performed on the Agilent BioAnalyzer and KAPA library quantification. Samples were sequenced on the Illumina Novaseq6000 S2 and data was packaged using the 10× Genomics SpaceRanger pipeline.

### Single nucleus RNA-seq

Since scRNAseq needs to be performed on fresh tissue, snRNAseq was chosen instead given its ability to be performed on previously frozen samples. snRNAseq samples were stored in liquid nitrogen for less than 5 months before processing. Nuclei were isolated using the Chromium Nuclei Isolation Kit (10X Genomics). snRNAseq was performed using the 10× Genomics Chromium X platform with 10× Genomics 3′ gene expression kit. Cell count and viability of the nuclei were verified on the ThermoFisher Countess 3 cell counter and EVO imager microscope. Single nuclei were parsed and proceeded into library preparation following manufacturer protocol. Quality control of the libraries was performed on the Agilent BioAnalyzer and Kapa library quantification. Samples were sequenced on the Illumina Novaseq6000 SP and data was packaged using the 10× Genomics CellRanger pipeline. The snRNAseq and spatial transcriptomic samples were patient-matched to increase the reliability of results during single-cell mapping and analysis.

### Quality control and data preprocessing

Data were imported into R and converted into Seurat objects. For quality control, nuclei with greater than 10% mitochondrial genes as well as nuclei with less than 200 genes in total were excluded from the analysis. These thresholds are consistent with published scRNAseq and snRNAseq data^30–32^. Mitochondrial, ribosomal, and heat shock protein genes were also excluded from the analysis^30^. To preserve biological variance present in each tissue sample, “SCTransform”, which implements regularized negative binomial models, was performed on each sample individually to normalize the data and detect high-variance genes (3000 high-variance genes were selected)^33^. The same quality control measures were implemented per spatial spot in the Visium data using Seurat. Any genes that were not measured in both the snRNA and Visium spatial transcriptomics were excluded from the analysis.

### Single nucleus analysis

snRNAseq data from normal and BA livers were integrated following the Seurat data integration pipeline (version 4.3.0)^10^. The number of features to return was set to 6000 in the “SelectIntegrationFeatures”, with “SCT” as the normalization method and “CCA” (canonical correlation analysis) as the reduction. Dimensionality reduction was performed after data integration using PCA (principal components analysis). Data clustering was performed using the Seurat “FindNeighbors” and “FindClusters” functions, using the original Louvain algorithm. The number of dimensions to be used during clustering was chosen using the elbow method (“ElbowPlot” function). The “k” parameter was chosen by taking the square root of the total number of nuclei in the integrated object. The resolution parameter was set to above 1 (2.5 to be precise) with the goal of overclustering and potentially identifying clusters of less frequent cell types. Gene expression heatmaps were used to aid in cluster and cell type annotation, which resulted in 24 subclusters (a total of 13 metaclusters). Proportions of each cell type were compared across samples and statistical significance was determined using permutation testing (1000 permutations; “scProportionTest”^34^).

### Spatial transcriptomics

Spatial samples were integrated following the Seurat data integration pipeline (version 4.3.0)^10^. The number of features to return was set to 3000 in the “SelectIntegrationFeatures”, with “SCT” as the normalization method and “CCA” (canonical correlation analysis) as the reduction. Differential gene expression (DE) was performed on spatial samples using the entire tissue slide to identify differences in gene expression between datasets. Seurat’s “FindAllMarkers” function was used to perform DE between clusters, regardless of group. Seurat’s “FindMarkers” function was used to perform DE to compare normal OCT vs SNAP and BA with advanced fibrosis OCT vs SNAP. Genes that were up- or downregulated in each comparison were identified and highlighted using a volcano plot. Pathway analysis was performed with pathfindR (version 1.6.4) using the DE output to further compare datasets, with KEGG as the gene set and using the greedy search algorithm to perform the active subnetwork search^35^. Clustering was done on the spatial samples to annotate the spatial spots following the same approach as with the snRNAseq samples, with a resolution value of 5.

### Single-cell spatial mapping using single-nucleus data

The annotated snRNAseq samples were used for single-cell spatial mapping using CellTrek (version 0.0.94)^17^. This process mapped individual annotated snRNAseq cell types to geographical locations within the tissue. To minimize false positives, the “top_spot” parameter was set to 5 (one nucleus can be mapped to at most 5 different spatial spots) and the “spot_n” value was set to 5 (one spatial spot can contain at most 5 nuclei). These parameter values—which are recommended by Wei et al.^17^—ensure fewer nuclei are being mapped to avoid over-estimation of the nuclei spatially. Increasing the number of mapped nuclei may increase the rate of false positives. At the same time, reducing these numbers to a 1:1 mapping of one nucleus per spot does not represent the true nature of the data (a spatial spot captures more than one cell/nuclei). Although from matched patients, the spatial and single nucleus data are still separate samples and forcing a 1:1 mapping would force all nuclei to be placed even if they are not the best match. The resulting labels were visualized on the tissues.

### Correlation analysis

Once the snRNAseq cell types were given spatial coordinates, CellTrek::scoloc was used to examine the colocalization of different cell types. This method calculates a 2D grid kernel density for each cell type and then uses Kullback–Leibler divergence to uncover global spatial patterns. Spatial relationships between different cell types where then visualized using the igraph R package (version 1.4.2)^36^.

### Ligand–receptor analysis

The CellChat (version 1.6.1) package was used to perform L–R analysis on both the snRNAseq data and the spatial transcriptomic data with mapped single-cell data^37^. These results were then compared between datasets and visualized on tissue sections.

### Statistical analysis

DE analysis was performed using a negative binomial generalized linear model and correcting for multiple testing using the Bonferroni correction method. Log2 fold-change of each gene comparison was calculated and plotted against its -log10(p-value) using a volcano plot. Genes with an absolute log2 fold-change greater than 1 and a significant p-value were considered significant and marked as either up or downregulated, depending on their log2 fold-change value. Spearman correlation analysis was used to compare L–R pairs that were downregulated in both, snRNAseq and spatial. A p-value < 0.05 was used to determine statistical significance. Seeds were set to allow for reproducibility. All statistical tests were carried out in R (version 4.2.2).

### Supplementary Information

Supplementary Figures.

## Data Availability

Data supporting the findings in this study have been made available on our Shinyapp (https://usctransplantlab.shinyapps.io/BAliver/). Raw primary data is available from the corresponding author upon reasonable request.

## Code availability

All code needed to analyze data and reproduce the results showed in this study will be made available on our lab’s Github (https://github.com/julietusc).
