# Supplementary material for: Technical optimization of spatially resolved single-cell transcriptomic datasets to study clinical liver disease
Source: Sci Rep. 2024 Feb 13;14:3612. doi: 10.1038/s41598-024-53993-2 (PMC10864257; doi:10.1038/s41598-024-53993-2)
Supplement: Supplementary file 1 — Supplementary Figures. [file 41598_2024_53993_MOESM1_ESM.pdf]

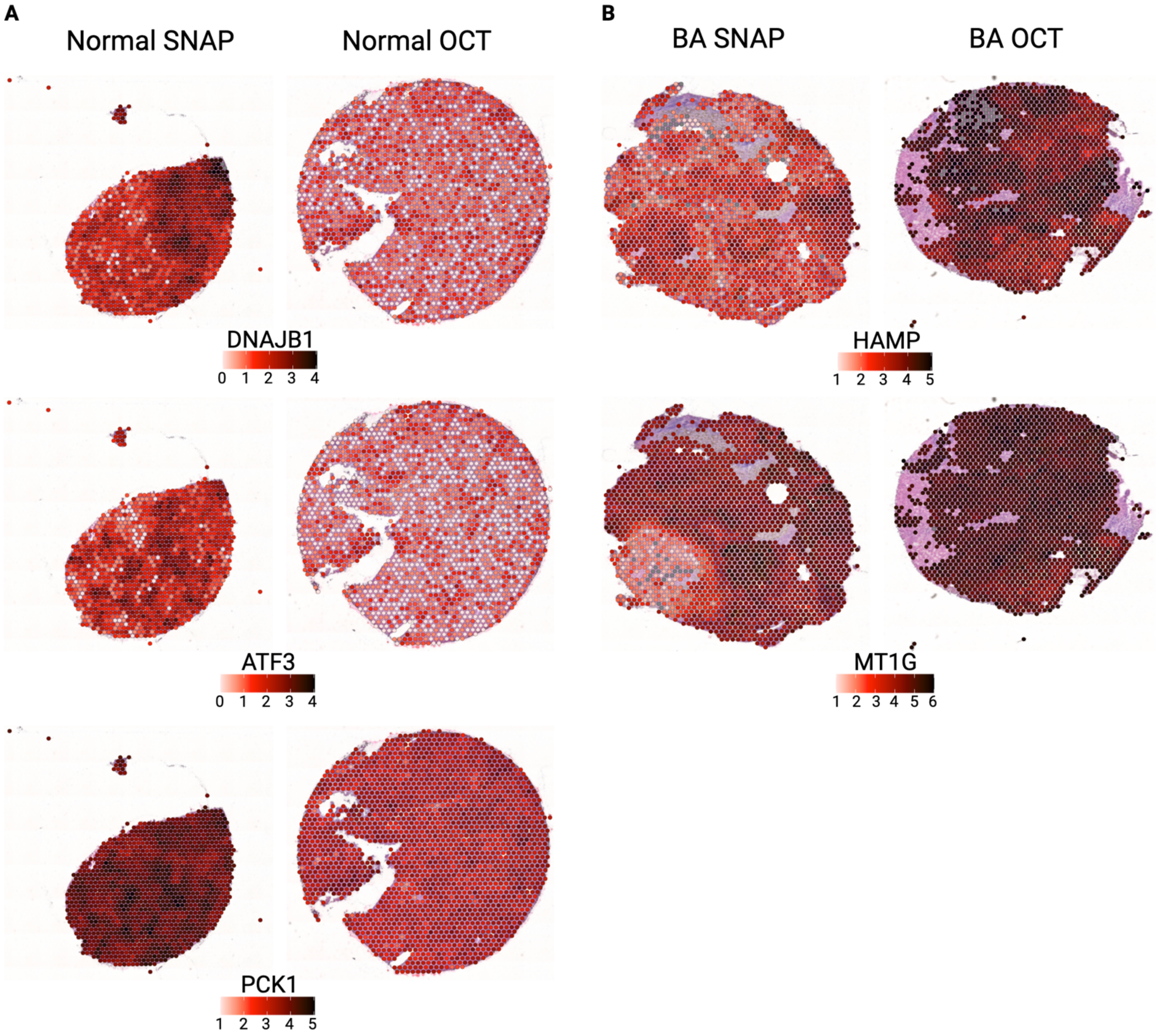

Supplementary Figure S1 | Liver tissue freezing technique's impact on differential gene expression when using spatial transcriptomic analysis is confirmed when visualizing gene expression on spatial sample. (**A**) Visual representation of differentially expressed genes in normal liver tissue shows upregulation of DnaJ heat shock protein family member B1 (DNAJB1), activating transcription factor 3 (ATF3), and phosphoenolpyruvate carboxykinase 1 (PCK1) in SNAP relative to OCT. (**B**) Visual representation of differentially expressed genes in BA liver tissue with advanced fibrosis shows downregulation of hepcidin antimicrobial peptide (HAMP) and metallothionein 1G (MT1G) in SNAP relative to OCT. The regions with absent cells represent regions with no spatial spots as they were discarded due to poor quality during our quality control steps. All figures were created in R (version 4.2.2).

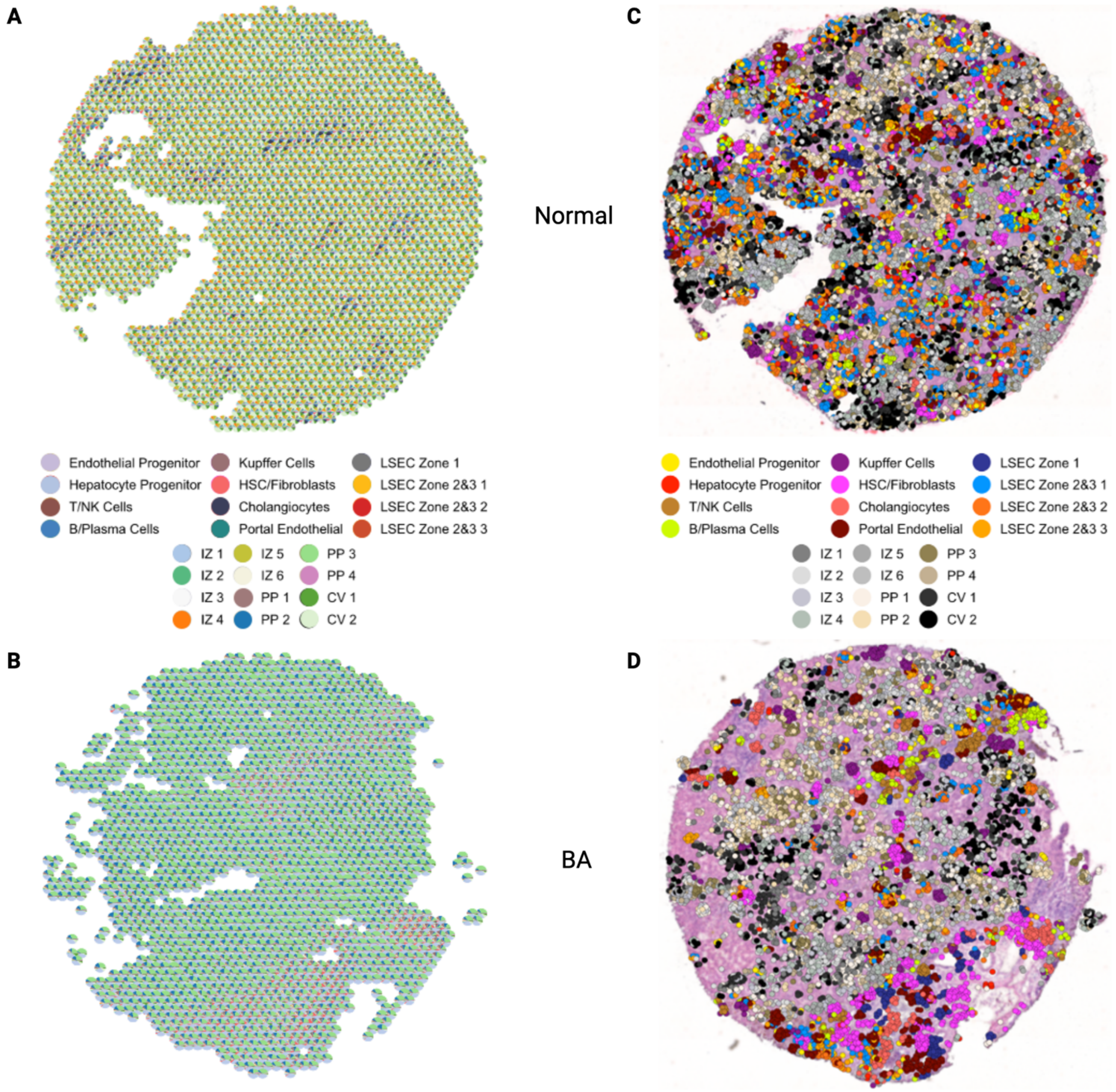

Supplementary Figure S2 | Comparison of techniques for integrating single-cell and spatial transcriptomics data demonstrates that mapping individual snRNASeq nuclei to Visium spatial transcriptomic 'spots' improves interpretability and downstream analysis in liver tissue. (**A,B**) Deconvolution of normal liver (A) and BA with advanced fibrosis (B) samples using CARD, a technique that maps cell type proportion to a single spot. Each Visium spot becomes a pie chart showing the percentage of different cell types in that spot. (**C,D**) Single-cell spatial mapping of normal liver (C) and BA with advanced fibrosis (D) samples using Celltrek, which maps individual cell types to specific Visium spots, creating one new spot for each individual nuclei. This technique makes it easier to interpret the output and use in any downstream analysis. The regions with absent cells represent regions with no spatial spots as they were discarded due to poor quality during our quality control steps. CV = central-venous hepatocytes. HSC = hepatic stellate cells. IZ = interzonal hepatocytes. LSEC = liver sinusoidal endothelial cells. NK = natural killer cells. PP = periportal hepatocytes. Figures a and b were created in CARD; figures c and d were created in R (version 4.2.2).

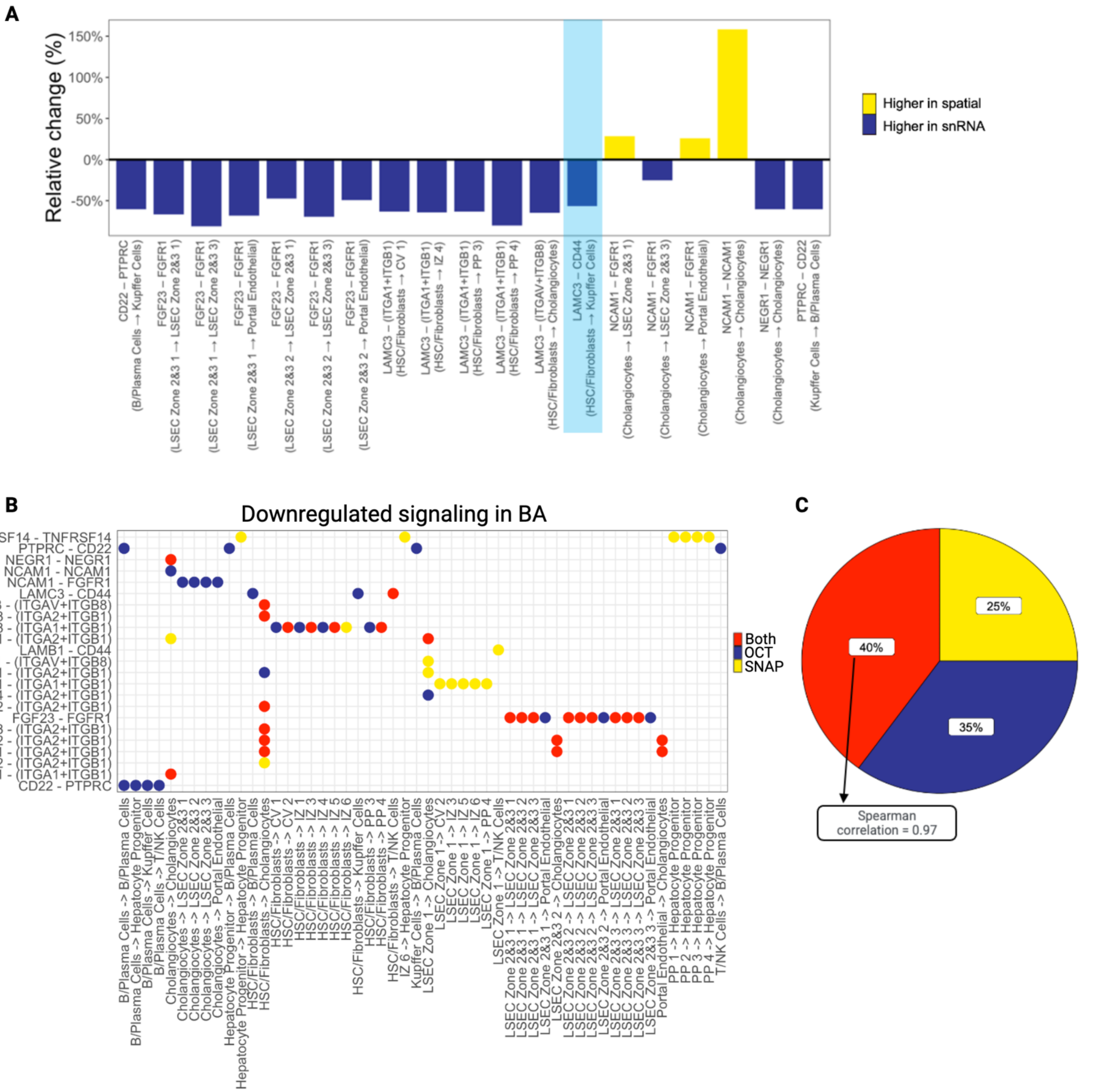

Supplementary Figure S3 | Ligand-Receptor analysis comparison between single-cell spatially mapped OCT and SNAP samples demonstrates high correlation of spatial interactions, while spatial and snRNAseq samples correlate poorly. (**A**) Relative change of communication strength in interacting cell types between resolved spatial transcriptomic data and snRNAseq data. Signal is lower in the spatially resolved data for most L-R signaling pairs. Highlighted in blue is the downregulated L-R signaling pair visualized in Fig. 6D. (**B**) L-R pairs in interacting cell types show a decrease in signaling in BA liver with advanced fibrosis when compared to normal liver tissue. The three distinct colors represent whether interactions were only downregulated in the spatially resolved OCT data (blue), only in the spatially resolved SNAP data (yellow), or in both datasets (red). (**C**) 40% of downregulated L-R signaling pairs are present in both spatially resolved datasets (Spearman correlation = 0.97). 25% of downregulated L-R signaling pairs are only present in the spatially resolved SNAP data, while 35% are only present in the spatially resolved OCT data. CV = central-venous hepatocytes. HSC = hepatic stellate cells. IZ = interzonal hepatocytes. LSEC = liver

sinusoidal endothelial cells. NK = natural killer cells. PP = periportal hepatocytes. All figures were created in R (version 4.2.2).
